# Supplementary material for: Exploration of mentor and mentee perspectives of a mentored clinical practice programme to improve patient outcomes in musculoskeletal physiotherapy
Source: PLoS One. 2022 Aug 26;17(8):e0272728. doi: 10.1371/journal.pone.0272728 (PMC9416989; doi:10.1371/journal.pone.0272728)
Supplement: Attachment — Submitted filename: Response to Reviewers.docx [file pone.0272728.s001.docx]

School of Healthcare Sciences

Cardiff University

Ty Dewi Sant

Heath Park

Cardiff

CF14 4XN

13^th^ June 2022

Dear Academic Editor and Reviewers,

Re: **Exploration of mentor and mentee perspectives of a mentored clinical practice programme to improve patient outcomes in musculoskeletal physiotherapy**

Thank you for your consideration of our above article and for your feedback on our original submission. We are pleased to respond to the points raised and to submit revised versions (marked and unmarked) of the manuscript in line with the corrections suggested.

*Line 51-53: I am not too sure about the advantage of using so many citations for a single argument/statement. Authors are advised to keep relevant citation/s only.*

Response: The point being made is the debate of this point over several decades, hence multiple sources being cited to support that multiple authors have been making this call over the decades.

*Line 55-60: The first sentence describing the results is not necessary when you have interpreted the results in the second sentence. The paragraph should also have a concluding remark.*

Response: Sentences edited and merged with concluding remark inserted for the paragraph.

*Line 65: Please explain what is M level.*

Response: explained in text.

*Line 83-98: It is difficult to understand which section describes the methods employed in this particular study. Please revise.*

Sentences revised and edited to clarify the methods employed.

*Line 110: Please explain what are these band levels.*

Band levels explained in text with additional reference.

*Line 213-214: Please check for grammar.*

Checked and corrected

*Line 819: Please remove.*

Removed

*Line 821-831: The discussion section should start with reiterating the objectives and key findings of the study.*

Initial paragraph inserted into the manuscript for this purpose.

*Quotations: All quotations should have line numbers given in the transcripts.*

*The manuscript is quite long. The results section should provide succinct findings and explanations/interpretations. Also, 1 quotation/observation note per theme should be enough.*

Quotation line numbers inserted. Multiple quotes removed to leave (usually) 1 quote per theme/subtheme to significantly shorten the length of the article.

We have also re-checked the references and style requirements as requested.

Thank you once again for your review and feedback. We do hope that the corrections are to your satisfaction.

Yours faithfully

Dr. Aled Williams (on behalf of the authors)
