# Supplementary material for: Exploration of mentor and mentee perspectives of a mentored clinical practice programme to improve patient outcomes in musculoskeletal physiotherapy
Source: PLoS One. 2022 Aug 26;17(8):e0272728. doi: 10.1371/journal.pone.0272728 (PMC9416989; doi:10.1371/journal.pone.0272728)
Supplement: Attachment — Submitted filename: Response to Reviewers.docx [file pone.0272728.s002.docx]

School of Healthcare Sciences

Cardiff University

Ty Dewi Sant

Heath Park

Cardiff

CF14 4XN

21^st^ July 2022

Dear Academic Editor and Reviewers,

Re: **Exploration of mentor and mentee perspectives of a mentored clinical practice programme to improve patient outcomes in musculoskeletal physiotherapy**

Thank you for your consideration of our above article and for your feedback on our original submission. We are pleased to respond to the points raised and to submit revised versions (marked and unmarked) of the manuscript in line with the corrections suggested.

*Line 84-95: Please describe your own study design NOT what has been employed in previous studies. You can add a separate paragraph on previous studies to support your methodology.

This comment also applies to participation selection and data collection and setting sections.*

Response: The text has been edited to state the study design, participant selection, data collection and settings at the beginning of the paragraphs before giving the justification for these decisions.

*Line 755: Why the heading 'discussion' has been deleted?*

Response: Heading has been reinserted.

*Line 65: Please explain what is M level.*

Response: explained in text.

*Line 759-762: Please add key findings of the study.*

Key findings have been added and summarised prior to discussion paragraphs.

Thank you once again for your review and feedback. We do hope that the corrections are to your satisfaction.

Yours faithfully

Dr. Aled Williams (on behalf of the authors)
